# Society Notes

**Published:** 1902-04

**Authors:** 


### SOCIETY NOTES.

THE Allegheny County Veterinary Medical Association, which is very active in its work and has nearly all the veterinarians of that bailiwick in its membership list, was recently addressed by Dr. A. Leteve, of the medical profession, on "Tetanus." Dr. Leteve is Director of the Pasteur Institute Department of the Magee Pathological Institute.

Dr. J. J. Hougendoubler, formerly located at Harrisburg, Pa., is employed as Assistant Inspector in Bureau of Animal Industry, St. Louis, Mo.

